# Inhibitory Control Over Ca^2+^ Sparks via Mechanosensitive Channels Is Disrupted in Dystrophin Deficient Muscle but Restored by Mini-Dystrophin Expression

**DOI:** 10.1371/journal.pone.0003644

**Published:** 2008-11-04

**Authors:** Martin D. H. Teichmann, Frederic v. Wegner, Rainer H. A. Fink, Jeffrey S. Chamberlain, Bradley S. Launikonis, Boris Martinac, Oliver Friedrich

**Affiliations:** 1 Medical Biophysics, Department of Systems Physiology, Institute of Physiology and Pathophysiology, Ruprecht-Karls-University, Heidelberg, Germany; 2 Department of Neurology, University of Washington, Seattle, Washington, United States of America; 3 School of Biomedical Sciences, University of Queensland, Brisbane, Queensland, Australia; Hospital Vall dHebron, Spain

## Abstract

**Background:**

In dystrophic skeletal muscle, osmotic stimuli somehow relieve inhibitory control of dihydropyridine receptors (DHPR) on spontaneous sarcoplasmic reticulum elementary Ca^2+^ release events (ECRE) in high Ca^2+^ external environments. Such ‘uncontrolled’ Ca^2+^ sparks were suggested to act as dystrophic signals. They may be related to mechanosensitive pathways but the mechanisms are elusive. Also, it is not known whether truncated dystrophins can correct the dystrophic disinhibition.

**Methodology/Principal Findings:**

We recorded ECRE activity in single intact fibers from adult wt, mdx and mini-dystrophin expressing mice (MinD) under resting isotonic conditions and following hyper-/hypo-osmolar external shock using confocal microscopy and imaging techniques. Isotonic ECRE frequencies were small in wt and MinD fibers, but were markedly increased in mdx fibers. Osmotic challenge dramatically increased ECRE activity in mdx fibers. Sustained osmotic challenge induced marked exponential ECRE activity adaptation that was three times faster in mdx compared to wt and MinD fibers. Rising external Ca^2+^ concentrations amplified osmotic ECRE responses. The eliminated ECRE suppression in intact osmotically stressed mdx fibers was completely and reversibly resuscitated by streptomycine (200 µM), spider peptide GsMTx-4 (5 µM) and Gd^3+^ (20 µM) that block unspecific, specific cationic and Ca^2+^ selective mechanosensitive channels (MsC), respectively. ECRE morphology was not substantially altered by membrane stress. During hyperosmotic challenge, membrane potentials were polarised and a putative depolarisation through aberrant MsC negligible excluding direct activation of ECRE through tubular depolarisation.

**Conclusions/Significance:**

Dystrophin suppresses spontaneous ECRE activity by control of mechanosensitive pathways which are suggested to interact with the inhibitory DHPR loop to the ryanodine receptor. MsC-related disinhibition prevails in dystrophic muscle and can be resuscitated by transgenic mini-dystrophin expression. Our results have important implications for the pathophysiology of DMD where abnormal MsC in dystrophic muscle confer disruption of microdomain Ca^2+^ homeostasis. MsC blockers should have considerable therapeutic potential if more muscle specific compounds can be found.

## Introduction

Duchenne muscular dystrophy (DMD) is a common inherited muscle disease. Death usually occurs early in adulthood from respiratory or cardiac failure. Different mutations in the dystrophin gene result in the complete absence of the 427 kDa protein dystrophin [1] that links the cytoskeleton to the extracellular matrix [2]. The mdx mouse, an animal model with an exon 23 point mutation, is extensively used to explore the biological functions of dystrophin [3]. The model was suitable to develop pharmacological [4]–[6] or gene therapy strategies to possibly treat the dystrophic phenotype [7], [8]. One approach is transfection with mini- or micro-dystrophin genes into mdx mice to stably express smaller dystrophin constructs [9].

Dystrophin plays a pivotal role in protecting the sarcolemma from mechanical, e.g. osmotic, stress [10] or contraction-induced damage [11], [12]. However, lately, the pathophysiological mechanism has become more complex than originally anticipated. Apart from ‘membrane hypotheses’ [13], dystrophin also affects ion channels [14]–[16], cellular Ca^2+^ regulation [17]–[19] and mechanotransduction [20], [21]. Although reports on elevated resting Ca^2+^ levels have been controversial [22]–[25], it has become more generally accepted that local Ca^2+^ homeostasis is disturbed [17], [26]. This may be mediated by Ca^2+^ influx through altered mechanosensitive channels [27], [28] that could contribute to increased Ca^2+^ dependent proteolysis [29], inflammation, repetitive degeneration/regeneration cycles and necrosis. Blockers of mechanosensitive channels (MsC) have been described to protect against muscle damage in the mdx mouse [30].

Elementary Ca^2+^ release events (ECRE) from sarcoplasmic reticulum (SR) in mammalian skeletal muscle were first demonstrated by our group in ‘*skinned*’ fibers [31] and since then used as a tool to study the molecular basis of ec-coupling. It has long been thought that, in intact fibers, the occurrence of ECRE is almost completely suppressed by some very tight DHPR-RYR coupling that was uncoupled in chemically or mechanically ‘*skinned*’ fibers [31]. Recently, however, Wang et al. [32] reported spontaneous ECRE in intact single wt and mdx flexor digitorum brevis (fdb) fibers that were challenged with hyper- or hypo-osmolar external solutions. Under isotonic conditions, no spontaneous ECRE were observed. It was concluded that osmotically-induced membrane deformations can induce ECRE activity in intact mammalian skeletal muscle in a reversible manner [32]. This ongoing activity in mdx fibers was suggested to act as a dystrophic signal. However, the precise molecular mechanisms by which it might be conferred from the membrane deformation to SR Ca^2+^ release were not resolved. Also, the authors performed their osmotic challenge experiments in hypertonic solutions containing unphysiologically high Ca^2+^ concentrations of 50 mM [32].

From the results of our present study, we propose a model in which altered MsC in dystrophic muscle modify the otherwise suppressed coupling between DHPR and RYR in intact resting fibers under isotonic conditions and even more so in response to membrane stress.

## Materials and Methods

### Muscle preparations, genetic strains, solutions

C57/SVJ129 wild-type (wt), mdx mice and mini-dystrophin (MinD) mice referring to a transgenic strain with the mdx background (CVBA3') that stably expresses a Δexon17-48 truncated mouse dystrophin of 228 kDa molecular weight [33] were used. After sacrifice of the animals by exposure to a 5% CO_2_-atmosphere, interossei muscles were quickly dissected. Killing of the animals and tissue preparation were according to guidelines of the local Animal Care Committee. Single fibers were obtained by mild enzymatical treatment in isotonic Ringer's solution that contained (mM): NaCl 140, KCl 5, CaCl_2_ 2, MgCl_2_ 1, Hepes 10, pH 7.4. Hypertonic 50 mM Ca^2+^ containing solution was composed as given by Wang et al. [32] and contained (mM): NaCl 140, KCl 4, CaCl_2_ 50, MgCl_2_ 1, Hepes 10, pH 7.4. As we consider its Ca^2+^ concentration as unphysiologically high, 300 mM sucrose was added to Ringer's solution (2 mM Ca^2+^, ∼550 mosM). A similar solution was previously used to record L-type Ca^2+^ currents in the strains [16]. Hypotonic solution was again identical to the one given by Wang et al. [32] (2 mM Ca^2+^, ∼120 mosM) and contained (mM): NaCl 70, KCl 4, CaCl_2_ 2, MgCl_2_ 1, Hepes 10, ph 7.4. Ca^2+^-free solutions were made without addition of CaCl_2_ and additionally contained 1 mM EGTA. To keep free Mg^2+^ concentration roughly constant, Mg^2+^ was increased to 2 mM in these solutions. Streptomycine and Gd^3+^ (Sigma Aldrich, Germany) and GsMTx-4 (Peptide Institute Inc, Osaka, Japan) were added from stocks to the desired concentrations. All experiments were performed at room temperature (22–24°C).

### Experimental procedure and image acquisition

Single fibers were stained with the fast high-affinity Ca^2+^ dye Fluo-4-AM (10 µM) in isotonic Ringer's solution for 20 min. Single fibers were transferred to the experimental chamber and mounted on the stage of a laser-scanning confocal microscope (FluoView 300, Olympus) equipped with a 20 mW Ar^+^/Kr^+^ ion laser (Omnichrome, Melles Griot). Excess external dye ester was washed out by exchanging the solution three times using a fast custom-built perfusion system that allowed complete solution exchange within 3 s. Intracellular, de-esterified Fluo-4 was excited at 488 nm and emission collected using a >515 nm low-pass filter. PMT voltage was between 630 V and 680 V between experiments. XYT image series were acquired at a sampling rate of 0.9 Hz with a 512×512 pixels resolution using a ×40 water immersion objective (NA 1.2, Olympus, Japan). XYT stacks contained up to 200 consecutive images (∼220 s total duration). XT line-scans were recorded at random line positions within the fibers. Repetitive acquisition time was 2 ms/line at a 512×1024 resolution, thus obtaining a full line-scan within two seconds. Different zoom factors between ×3 and ×7 were used and the adjacent voxel sizes used for calculation of Ca^2+^ spark dimensions.

The following experimental procedures were carried out. First, elementary Ca^2+^ release events in the intact single fibers were recorded in isotonic Ringer's solution. Subsequently, XYT series and several XT line-scans were recorded (control). Then, fibers were either subjected to hypo- or hyperosmolar stress by quickly exchanging the isotonic Ringer solution to the appropriate solution of given osmolarity (see above). The solution exchange was performed while recording an XYT series to follow the time course of osmolarity effect on ECRE frequency. After a steady-state was reached, the solution was changed back to isotonic Ringer's solution to test for reversibility of the observed osmolarity induced changes. During solution exchange, movement artefacts of the single fibers were frequently observed. As the solution exchange was complete within 3 s, this corresponded to two subsequent images in the recording sequence only. Therefore, in the corresponding recordings, the images containing movement artefacts were discarded from ECRE analysis, as they gave abrupt changes in ECRE frequency resulting from false positive events during the fiber movement. In a subset of experiments evaluating the contribution from extracellular Ca^2+^ to the ECRE frequency during osmotic stress, ECRE were first recorded in isotonic Ca^2+^ containing Ringer's solution, then solution exchanged to hypo-/hyperosmolar Ca^2+^ free solution while recording and, finally, back to isotonic Ca^2+^ containing Ringer's solution. To test the involvement of mechanosensitive channels to the osmotic stress induced changes in ECRE, different blockers of mechanosensitive channels (MsC) were used: streptomycine (200 µM), an unspecific blocker of MsC [28], Gd^3+^ (20 µM) a blocker of more Ca^2+^ sensitive MsC [28], [34], [35] and the recently synthesized specific inhibitor of cation-selective MsC GsMTx-4 (5 µM) derived from tarantula spider toxin [36]. To differentiate between the possibilities that drugs either potentially block or prevent increased spark activity in mdx fibers following osmotic stress, two different settings were designed and tested in mdx fibers: (i) from isotonic Ringer's solution, osmotic stress was induced by solution exchange to hypo-/hypertonic Ca^2+^ containing solution and either drug applied after spark frequency increased in response to the osmotic stimulus. This was suitable to assess the blocking potency of the drugs on osmotic stress-induced ECRE frequencies. (ii) ECRE were recorded in Ringer's solution that was then pre-incubated with either streptomycine or Gd^3+^ and solution was then exchanged to hypo-/hyperosmolar Ca^2+^ containing external solution also containing the drug. This was suitable to test whether both drugs prevented uncontrolled spark activity in mdx fibers by osmotic stress. Both experimental approaches were carried out in 2 mM Ca^2+^ containing solutions. In some experiments, (i) was performed in mdx fibers applying GsMTx-4 after osmotic challenge under Ca^2+^-free external conditions throughout.

### Image and data analysis

XYT and XT images were background normalized and denoised using the á trous implementation of the two-dimensional undecimated discrete wavelet transform (DWT) as previously described [37], [38]. Briefly, an estimate of background fluorescence was obtained from the mean of all images within a series. Randomly appearing ECREs are reliably attenuated in this mean image whereas still structures, e.g. sarcomere patterns, are conserved. Each image within the series was then divided by the mean image to remove t-tubular patterns and a cut-off criteria was applied setting all pixel values to zero that exceeded a 1.5 σ threshold above the mean (i.e. F/F_0_+1.5 σ). A second run for background estimate was then performed on this set of images with probable ECRE removed and all raw images were finally divided by this improved mean image, yielding normalized F/F_0_ images of a series. The á trous implementation contains the cubic B-spline as scaling function and a discrete low-pass filter (5-tab binomial filter, [39]). The denoising parameter δ controlling the amount of wavelet coefficients classified as noise within each wavelet scale was set to 3 [38]. Noise removal was applied using a hard-thresholding technique, i.e. setting subthreshold pixels to zero and finally, the denoised image was re-constructed as given by Conti et al. [40]. Event detection of ECRE was performed on the DWT of the denoised images within a series using the same detection parameters as described before [38]. ECRE events were counted in each image and normalized with respect to time (‘*spark frequency SF*’, ECRE/s). Additionally, ECRE frequency was normalized with respect to fiber cross-sectional area (*‘spatial spark frequency SSF’*, ECRE/s/100 µm^2^) using the voxel size at a given zoom factor. For osmotic challenge, up to ∼380 individual fibers were scanned and analysed for each strain and condition. The imaging algorithm yielded the overall average SF or SSF for a given XYT series. To quantify the time course and kinetics of ECRE frequency changes upon solution exchange, the SF or SSF were analysed on a frame by frame basis. The apparent SF and SSF values are obviously larger than those obtained for the whole time series, simply due to averaging. ECRE kinetics were analysed in 15 randomly selected fibers from each strain under conditions of solution exchange to either hypertonic or hypotonic 2 mM Ca^2+^ containing external medium (experiments without blockers). The time-to-peak (TTP) from the exchange of solution to the maximum of SSF induced by hypertonic or hypotonic medium and the mean apparent peak SSF values (SSF_max_) were analysed. Under maintained osmotic challenge, apparent SSF following osmotic shock showed adaptation with spontaneous decay of SSF. This decay was fitted by a single exponential with a time constant τ_dec_ for the decay kinetics. In experiments testing different MsC blockers, XYT series from all fibers were analysed on a frame-by-frame basis extracting the apparent SSF_max_ values and aligned so that blocker applications were at the same time point for all fibres tested per blocker and condition.

In line scan images (XT), the ECRE morphology was determined as given by v Wegner et al. [37]. ECRE amplitudes, full-duration at half-maximum (FDHM), full-width at half-maximum (FWHM) and rise-time (RT) were computed. All imaging procedures were performed using a self-written imaging algorithm using IDL 6.0 software (IDL research systems, Boulder, CO). Data are given as mean±SEM. n refers to ECREs in XT-scans and to fibers in XYT-scans. Student's paired t-test was performed to compare osmotic effects within the same fibers. Unpaired t-tests or ANOVA were performed between groups of strains. Paired t-tests were performed to assess drug effects in the same fibers. P<0.05 was considered significant. For preparation of figure images, data images were post-processed using Image J (http://rsb.info.nih.gov/ij/). Specifically, LUTs and a median filter (2.0 pixels) were applied for visualization purposes only.

### Resting membrane potential recordings in intact muscle fibers

Whole interossei muscles from wt and mdx mice were exposed from the paw and placed in a recording chamber containing either isotonic or hypertonic (300 mM sucrose) Ringer's solution (2 mM Ca^2+^). An equilibration time of 30 min was allowed before commencing potential recordings. Resting membrane potentials were recorded using a GeneClamp 500 amplifier (Axon Instr., Foster City, USA). Sharp microelectrodes were pulled from borosilicate capillaries using a horizontal puller (P87, Sutter Instruments) and filled with 3 M KCl. Microelectrodes were mounted on a pipette holder and connected to the headstage of the amplifier. The headstage was held by a manual micromanipulator to allow fine movements of the pipette tip. Tip resistances were between 5 MΩ and 10 MΩ. The approach using exposed muscles ensured recordings to be made as close as possible to the physiological condition without further unspecific damage during dissection and subsequent enzymatic treatment. The latter may otherwise result in a larger proportion of depolarised fibres, thus skewing the real membrane potential distributions especially in the more fragile mdx muscle. After offsetting the potential readings (0 mV), the muscle was slowly impaled with the tip of the microelectrode until a negative potential deflection was noticed. The most negative value during this deflection was taken as the resting membrane potential of an individually impaled single fiber. The pipette was then pulled back and impalement was repeatedly performed after random repositioning of the electrode over the muscle flap. Impalements with a potential drift >5 mV after withdrawal of the electrodes were discarded. In experiments assessing the contribution of mechanosensitive channels to the resting membrane potentials, the specific MsC blocker GsMTx-4, a peptide contained in the venom of the Chilean Rose Tarantula, *Grammostola spatulata*, was used. The peptide was added at a concentration of 5 µM to the isotonic or hypertonic recording solution before the challenge of the contralateral muscle from the animal of which the corresponding recordings were done in the absence of GsMTx-4 that day.

## Results

### Ca^2+^ sparks in resting intact single wt, mdx and MinD muscle fibers under isotonic conditions

**Figure 1:**
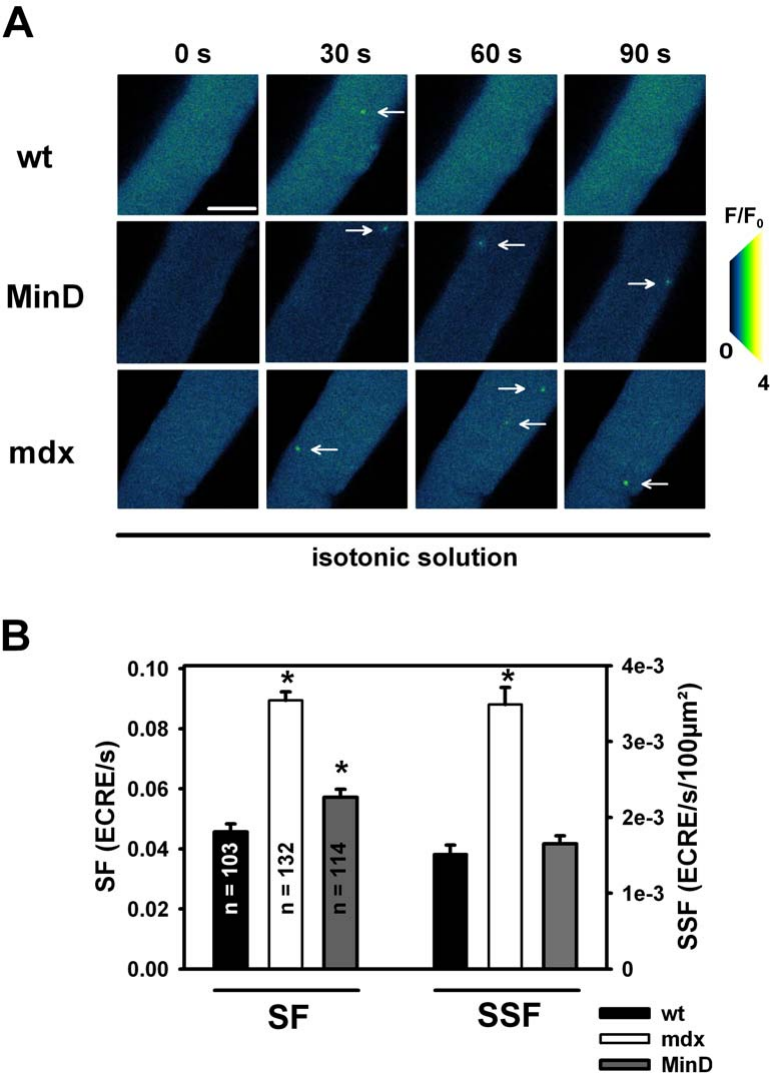
ECREs in intact single muscle fibers from wt, mdx and MinD mice under isotonic conditions. A, image sequence from a time series of ECRE (arrows) recordings in a resting intact wt, mdx and MinD fiber bathed in isotonic external solution. B, ECRE are more frequent in mdx fibers than in wt or MinD fibers. SF: spark frequency. SSF: spatial spark frequency. *: P<0.05 vs. wt. Scale bar: 40 µm.

ECRE recordings under physiological isotonic conditions (2 mM external Ca^2+^) in intact single wt, mdx and MinD fibers, as shown in Fig. 1A, suggest a larger ECRE frequency in resting mdx fibers. This is confirmed in over 100 single fibers investigated within each strain (Fig. 1B). Resting isotonic ECRE frequencies (SF; cross-sectional area normalized: SSF) are highly significantly increased in mdx fibers compared to wt fibers (P<0.0001) whereas in MinD fibers they were still large but more similar to wt fibers (table 1, Fig. 1B).

**Table 1:** SSF in single wt, mdx and MinD fibers in isotonic, hypertonic and hypotonic solution.

strain	osmotic condition	Ca^2+^ concentration	SSF (10^−3^ ECRE/s/100 μm^2^)	n
**wt**	isotonic	2 mM Ca^2+^	1.51±0.12	103
	hypertonic	50 mM Ca^2+^	11.38±0.56*	60
		300 mM sucrose, 2 mM Ca^2+^	6.80±0.68*	68
	hypotonic	2 mM Ca^2+^	5.71±0.31*	73
**mdx**	isotonic	2 mM Ca^2+^	3.48±0.23#	132
	hypertonic	0 mM sucrose, 50 mM Ca^2+^	20.04±0.67* ^,^ #	88
		300 mM sucrose, 2 mM Ca^2+^	18.13±0.55* ^,^ #	378
		300 mM sucrose, 0 mM Ca^2+^	13.57±0.35*	98
	hypotonic	2 mM Ca^2+^	17.16±0.82* ^,^ #	238
		0 mM Ca^2+^	13.70±0.52*	91
MinD	isotonic	2 mM Ca^2+^	1.65±0.11	114
	hypertonic	50 mM Ca^2+^	9.31±0.33* ^,^ #	52
		300 mM sucrose, 2 mM Ca^2+^	5.42±0.33* ^,^ #	118
	hypotonic	2 mM Ca^2+^	3.82±0.26* ^,^ #	87

* P<0.001 vs. isotonic same strain.

# P<0.05 vs. wt, same osmotic challenge.

### Osmotic challenge triggers increased ECRE activity in wt, mdx and MinD fibers

**Figure 2:**
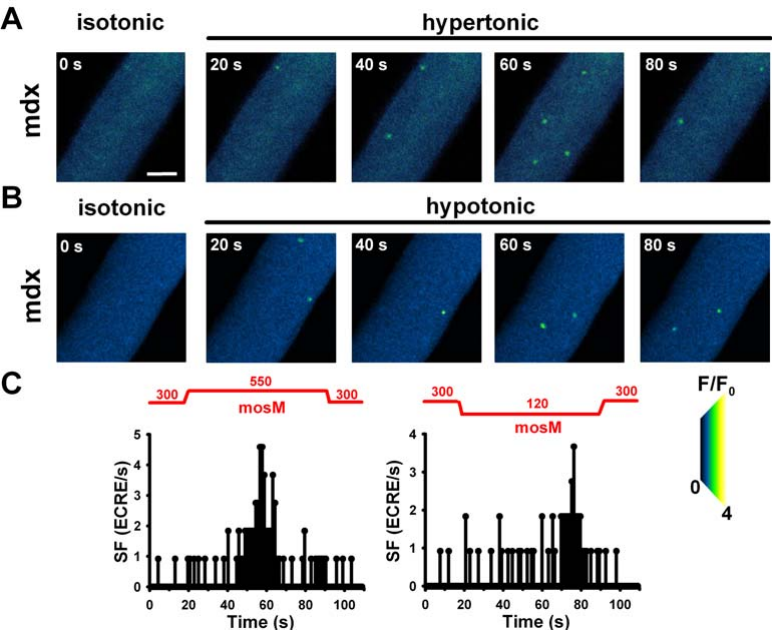
ECRE activity following hyperosmolar or hypoosmolar challenge. Image series from a single intact mdx fiber either stressed with hypertonic (A) or hypotonic external solution (B). Compared with the initial isotonic condition, osmotic challenge markedly increases apparent SF (C). The response time for the SF increase in hypotonic solution is larger than in hypertonic solution. Apparent SF shows adaptation to ongoing osmotic challenge with a rapid decline before the washout of hyper-/hypotonic medium. Scale bar: 20 µm.

**Figure 3:**
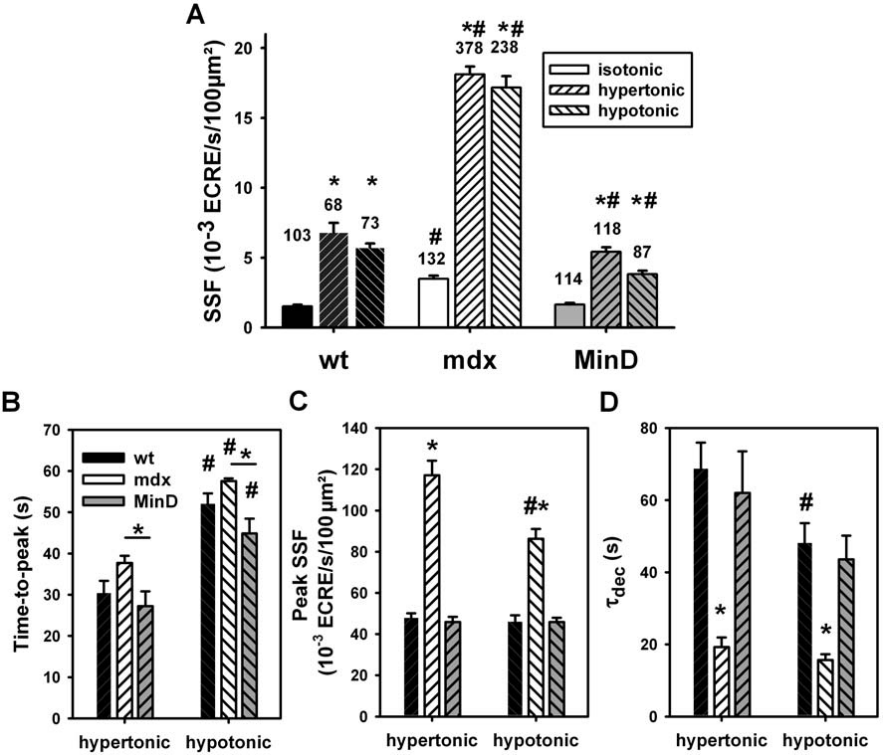
ECRE activity increase and kinetics by hypertonic and hypotonic stress. A, SSF evaluated from XYT recordings in several hundred (n) single wt, mdx and MinD fibers under isotonic, hypertonic or hypotonic external conditions. Average SSF values are similar in wt and MinD fibers but significantly increased in mdx fibers under all conditions. The kinetics of the apparent SSF based on a frame-by-frame analysis is shown in Fig. 2C for a random selection of 15 fibers for each condition and strain. (B), (C) and (D), time-to-peak (TTP), peak SSF and time constants τ_dec_ of the exponential SSF decline under maintained osmotic challenge. *: P<0.05 vs. both wt and MinD, or as indicated by the appropriate bar. #: P<0.05 hypotonic vs. hypertonic, same strain.

External osmotic shock (hypertonic; 50 mM external Ca^2+^) resulted in ‘uncontrolled’ spark activity in mdx fibers [32]. We repeated such recordings (table 1), but also kept external Ca^2+^ concentrations at physiological levels (i.e. 2 mM). Under such conditions, there was a clear increase in ECRE activity following osmotic challenge (Fig. 2A, B). The time course of ECRE response is shown in Fig. 2C by the apparent SF values in successive images. The SF increase following osmotic challenge had a larger phase lag for hypotonic compared to hypertonic solution (time-to-peak for the fibers shown: 40 s hypertonic, 59 s hypotonic). Even before washout, SF slowly returned to initial values suggesting an inactivation of the increased ECRE activity. Fig. 3A and table 1 summarize mean averaged SSF values from several hundred wt, mdx and MinD single fibers. For both hypertonic and hypotonic challenge, SSF was significantly increased in each strain compared to resting isotonic condition. The differences in ECRE kinetics following either hypertonic or hypotonic challenge were statistically verified in a random selection of 15 fibers for each strain and condition (Fig. 3B, C; table 2). TTP is consistently larger for all strains in hypotonic vs. hypertonic challenge (P<0.001). Peak SSF values were similar in wt and MinD fibers under all conditions but at least twofold increased in mdx fibers (P<0.001). Interestingly, in contrast to the conditions presented by Wang et al. [32], fibers in the present study never showed unterminated (‘uncontrolled’) ECRE activity but an exponential decline during ongoing osmotic challenge (Fig. 2C) with a time constant τ_dec_ (Fig. 3D, table 2). τ_dec_ was always similar in wt and MinD fibers. In mdx fibers, SSF declined two- to three times faster in both hypertonic and hypotonic solution. In 50 mM Ca^2+^ hypertonic solution, peak SSF was larger than in 2 mM Ca^2+^ hypertonic solution (table 1) and ECRE activity still declined with ongoing osmotic challenge, however, with a three to fivefold slower time constant in 50 mM Ca^2+^ hypertonic solution when compared to 2 mM Ca^2+^ or even Ca^2+^-free solution (table 2).

**Table 2:** Peak SSF, time-to-peak (TTP) and time constant of SSF decline for maintained osmotic challenge, τ_dec_, in wt, mdx and MinD fibers.

strain	Ca^2+^	hypertonic			hypotonic		
		TTP (s)	Peak SSF (10^−3^ ECRE/s/100 μm^2^)	τ_dec_ (s)	TTP (s)	Peak SSF (10^−3^ ECRE/s/100 μm^2^)	τ_dec_ (s)
**wt**	2 mM Ca^2+^	30.4±2.9	47.9±2.1	68.7±7.2	52.0±2.5#	46.1±3.0	48.2±5.5#
**mdx**	2 mM Ca^2+^	37.7±1.4*	117.1±7.0*	19.3±2.7*	57.6±0.6* ^,^ #	86.3±4.8* ^,^ #	15.7±1.6*
	0 mM Ca^2+^	10.5±0.5*	77.6±0.6*	11.7±1.1*	14.9±0.6* ^,^ #	81.9±0.6*	9.1±0.7*,#
	50 mM Ca^2+^	24.9±4.9	106.9±0.7*	60.0±8.5	n.a.	n.a.	n.a.
MinD	2 mM Ca^2+^	27.3±3.6	45.9±2.4	62.0±11.5	44.9±3.5#	45.9±2.1	43.6±6.6

* P<0.05 vs. wt.

# P<0.05 hypotonic vs. hypertonic in the same strain.

**Figure 4:**
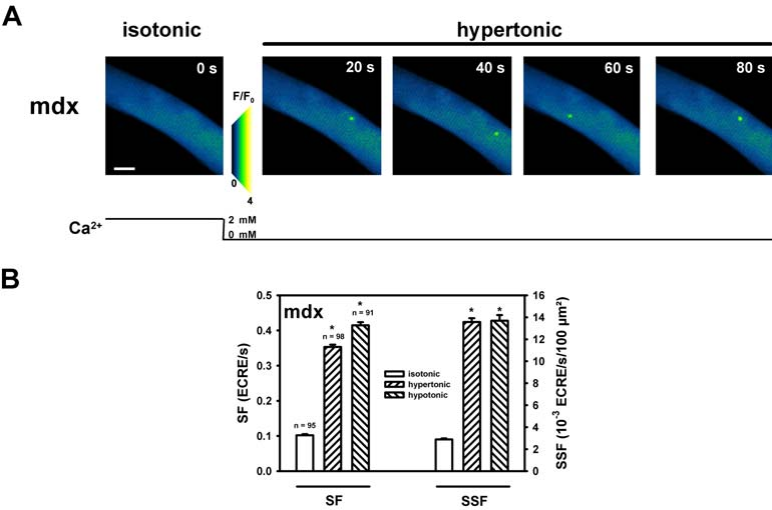
The increase in ECRE activity following osmotic challenge is independent of external Ca^2+^. A, representative recording of ECRE in a single mdx fiber under resting condition in isotonic solution and following osmotic stress in hypertonic Ca^2+^ free external solution at the time points indicated. B, summary of SF and SSF values from up to ∼100 single mdx fibers following hypertonic or hypotonic challenge without external Ca^2+^. The approximately fivefold increase in SSF under both conditions is similar to the increase observed in the presence of 2 mM external Ca^2+^ (Fig. 3A). *: P<0.001 vs. isotonic solution. Scale bar: 25 µm.

We next reasoned whether external Ca^2+^ was necessary for the increase in ECRE activity induced by osmotic shock. We, therefore, performed osmotic challenge experiments in the absence of external Ca^2+^ in mdx fibers where the increase in SSF following osmotic shock was most pronounced. Fig. 4A shows a section from a XYT recording in a representative single mdx fiber under isotonic resting conditions (2 mM external Ca^2+^) and following osmotic challenge in Ca^2+^-free hypertonic solution at the time points indicated. The marked increase in SF and SSF was confirmed in up to 100 single mdx fibers, as shown in Fig. 4B and table 1. The almost five-fold increase in SSF following osmotic challenge in absence of external Ca^2+^ parallels a similar, albeit somewhat smaller, increase as compared to the presence of 2 mM external Ca^2+^ and, thus, rules out the necessity of (but a regulation by) external Ca^2+^ to markedly increase ECRE activity in mdx fibers.

### Increased ECRE activity during osmotic challenge is mediated by mechanosensitive signalling pathways that are disrupted in mdx fibers

**Figure 5:**
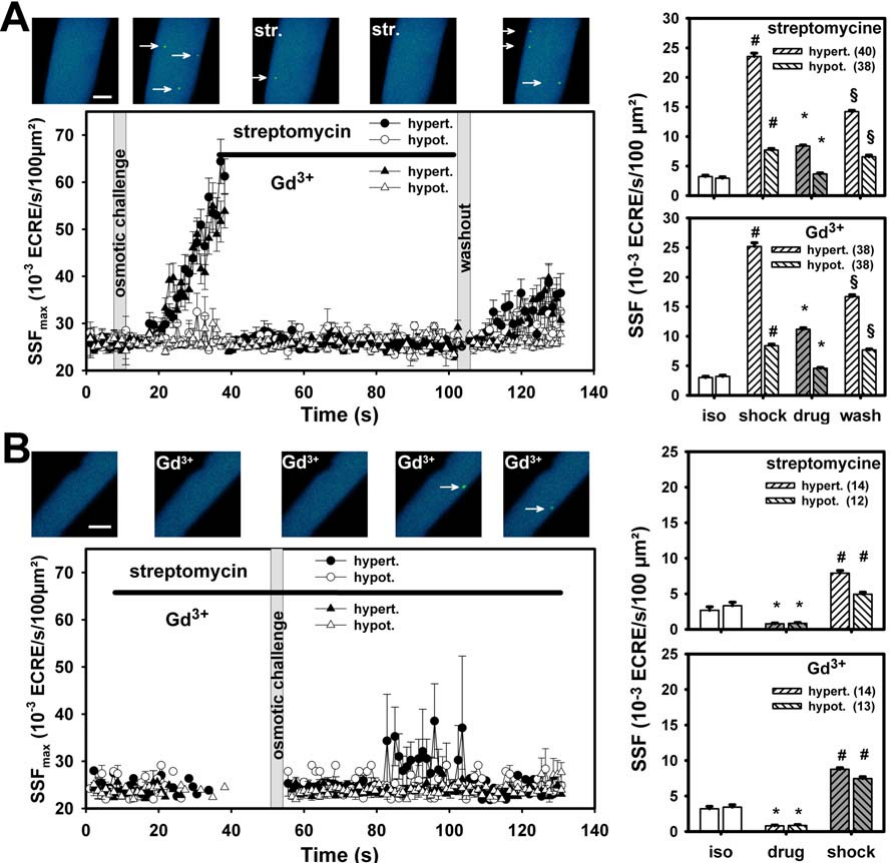
Osmotic-induced increase in ECRE activity is mediated by mechanosensitive channels. A, mean apparent ECRE frequencies (SSF_max_) on a frame-by-frame analysis from ∼40 single mdx fibers following osmotic challenge (hypertonic: filled symbols, hypotonic: open symbols) and application of either 200 µM streptomycine (circles) or 20 µM Gd^3+^ (triangles), respectively. The presence of the blockers is indicated by a black line. Image sequence above: ECRE response in a representative single mdx fiber during hypertonic shock and streptomycine (str.) application. Right two bar panels show averaged SSF values during the time intervals prior to osmotic shock (isotonic), during osmotic shock, after blocker application and during washout. B, SSF_max_ values in at least twelve single mdx fibers subjected to osmotic shock after pre-incubation with the MsC blockers. Pre-incubation with blockers failed to induce the marked response of osmotic shock seen in (A). Discontinuities in SSF_max_ are due to the fact that data points were omitted for clarity when no ECREs were present in all fibers analysed for this time point (SSF_max_ = 0). All experiments performed in 2 mM Ca^2+^ containing external solution. Image sequence above: fiber pre-incubated with Gd^3+^ followed by hypotonic shock. *: P<0.01 blockers compared to the previous condition, #: P<0.001 osmotic shock compared to the previous condition, §: P<0.01 washout is different from all previous conditions. Scale bars: 20 µm.

Swelling or shrinking during osmotic shock can be associated with changes in tubular membrane tension [41] although tubular distances remain relatively constant upon stretching [42]. To test whether the osmotic-induced increase in SSF was mediated by mechanosensitive channels, the time course of apparent SSF was recorded in ∼40 single mdx fibers either subjected to hypotonic or hypertonic solution (2 mM Ca^2+^), followed by addition of either streptomycine (200 µM) or Gd^3+^ (20 µM). Note that their mean values SSF_max_, shown in Fig. 5A, are dominated by fibers that present ECRE activity at a given time point. For visualization purpose only, zero SSF_max_ values were omitted from the plot, thus, producing some discontinuity (Fig 5B). SSF_max_ markedly increased following osmotic shock, more pronouncedly following hypertonic challenge. Application of both blockers abruptly terminated the high ECRE activity. After washout of the drug (osmotic challenge still present) ECRE activity increased again but with a rate lower than that compared to the increase before blocker application. To test whether the blockers could prevent the osmotic-induced increase in ECRE activity, osmotic shock was initiated after pre-incubation of either blocker in isotonic solution and maintained in the non-isotonic solution (Fig. 5B). Both blockers prevented a marked increase in SSF_max_. Gd^3+^ was more potent than streptomycine. In the presence of blockers, elevated averaged SSF levels in mdx fibers in isotonic solution were significantly reduced to levels even below those ones found in resting wt fibers. Although the osmotic shock-induced increase in ECRE activity following pre-incubation with either blocker was still significant, it was markedly less than in the absence of blockers.

**Figure 6:**
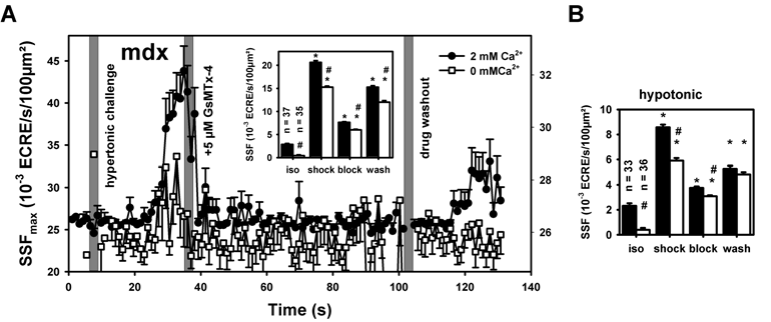
Ca^2+^ dependent and Ca^2+^-independent component of MsC-mediated osmotically induced ECRE activity. A, apparent SSF_max_ values during hypertonic challenge in ∼35 single mdx fibers following hypertonic challenge in either 2 mM Ca^2+^ containing (filled circles) or Ca^2+^-free external solution (open squares) and block of ECRE responses by application of 5 µM of the specific MsC blocker GsMTx-4. The inset shows the averaged SSF values during the time intervals in isotonic solution, during hypertonic shock, after blocker application and during washout. Note the different scale for both external Ca^2+^ conditions. B, shows the averaged SSF values from similar experiments performed in ∼35 other single fibers during hypotonic shock. *: P<0.001 compared to the previous conditions. #: P<0.001 Ca^2+^-free vs. 2 mM Ca^2+^ under the same condition (isotonic, shock, block or washout).

As seen before (table 1, 2; Fig. 4), there seems to be a Ca^2+^ dependent and a Ca^2+^ independent contribution to the ECRE increase in osmotically challenged fibers. To separate these contributions specific to MsC activation upon osmotic challenge, the specific inhibitor of stretch-activated cation channels in muscle, GsTMx-4 (5 µM) was used. Fig. 6A shows results from recordings in ∼40 single fibers similar as in Fig. 5A with GsMTx-4 applied after ECRE increases in response to hypertonic challenge either in 2 mM Ca^2+^ containing or Ca^2+^-free environment. GsMTx-4 potently terminated the increased ECRE activity even if external Ca^2+^ was absent. Averaged SSF values (inset in Fig. 6A) were much lower already in Ca^2+^ free isotonic starting conditions (inset) highlighting Ca^2+^ influx already being present in mdx fibers under isotonic conditions [28]. Fig. 6B shows the averaged SSF values from similar recordings using GsMTx-4 under hypotonic shock conditions that also potently blocked the ‘uncontrolled’ spark activity after osmotic challenge.

### ECRE morphologies in osmotically stressed single mdx fibers

**Figure 7:**
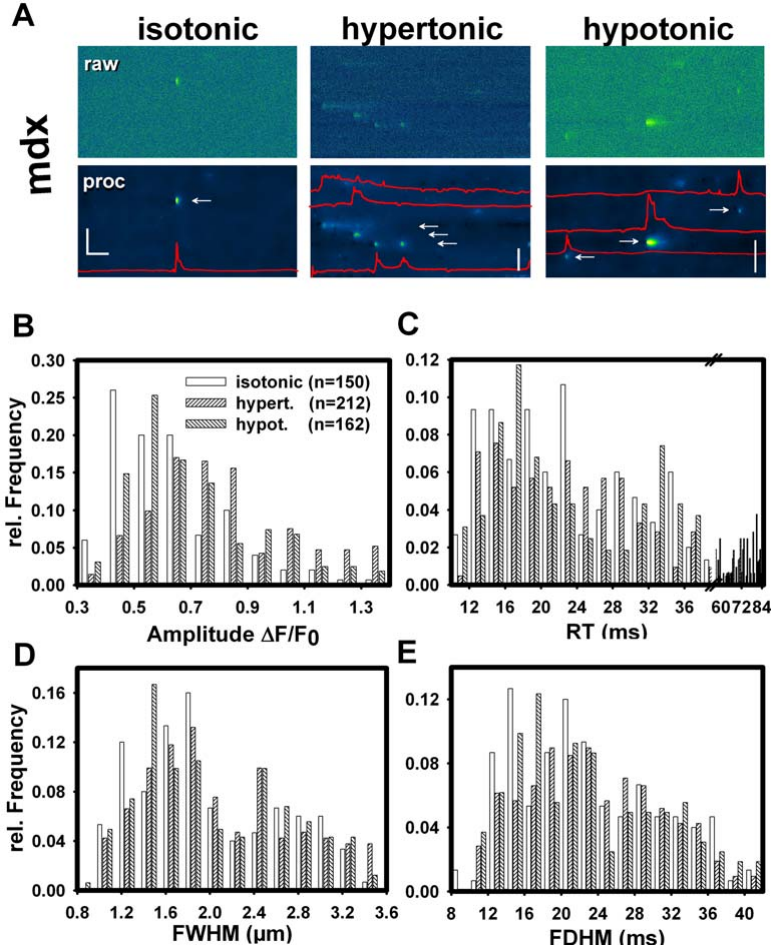
ECRE morphology in single mdx fibers following osmotic challenge. A, line-scan (XT) recordings in a single mdx fiber under isotonic, hypertonic or hypotonic external conditions. Upper row: noisy raw data; lower row: wavelet-denoised data. ECRE time course is shown by intensity profiles (red lines). Morphology parameter histograms from several hundred ECREs: ECRE amplitudes (B), rise-times RT (C), full-width at half-maximum FWHM (D), full-duration at half-maximum FDHM (E). Apart from a right-shift of the amplitude and RT distributions, there were no major alterations in ECRE morphology following osmotic challenge. Horizontal scale bar: 200 ms, vertical scale bars: 20 µm.

To clarify whether ECRE morphology was altered by osmotic challenge in intact mdx fibers, line-scans (XT) were recorded (Fig. 7A). The increase in ECRE frequency following osmotic challenge was again observed. ECRE spatial and temporal characteristics from several hundred events are summarised in Fig. 7B–E. Amplitude histograms were right-shifted after osmotic challenge, as confirmed by comparing the cumulative binned frequency distributions for the amplitudes (not shown). This amplitude right-shift was even more pronounced for hypertonic compared to hypotonic challenge (half cumulative frequency values CF_50_: 0.73 (hypertonic), 0.58 (hypotonic) and 0.53 (isotonic)).

### Ca^2+^ waves induced by osmotic challenge in mdx fibers

**Figure 8:**
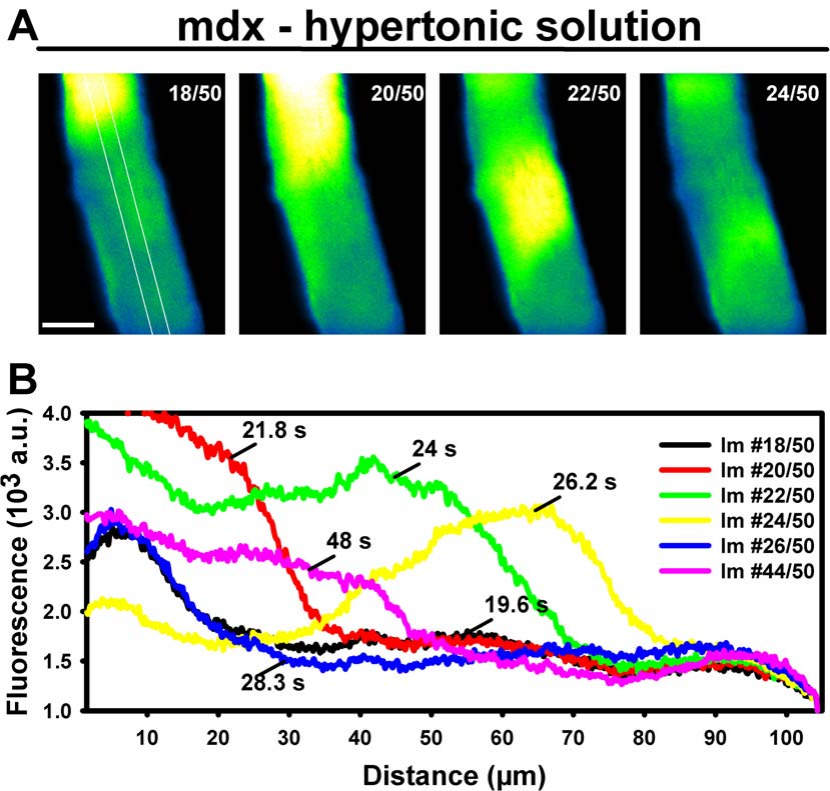
Ca^2+^ waves induced by osmotic challenge in single mdx fibers. A, single mdx fiber with global Ca^2+^ waves rather than increased ECRE activity following osmotic challenge (hypertonic solution, 2 mM Ca^2+^). Numbers indicate the frame number during a 50 frame XYT series. Scale bar: 20 µm. White line: ROI from which spatial profiles (B) were obtained. Ca^2+^ waves were never observed in wt or MinD fibers.

In some mdx fibers (hypertonic challenge: 7.1%, hypotonic: 6.7%), global Ca^2+^ waves could be detected that spread forth and backwards on a seconds time scale (Fig. 8A, Movie S1) and seemed to repetitively originate from distinct areas near the fiber ends (Fig. 8B). Such Ca^2+^ waves were never observed in any wt or MinD fiber subjected to osmotic challenge. Another observation made in a subset of fibers was a twofold homogenous increase in resting background fluorescence over ∼2 min in both hypertonic (six out of 68 wt, 37 out of 378 mdx, 10 out of 118 MinD fibers, P = 0.93, χ^2^-test) and hypotonic 2 mM Ca^2+^ containing solution (five out of 73 wt, 22 out of 238 mdx, seven out of 87 MinD fibers, P = 0.8).

### Involvement of MsC to resting membrane potentials in osmotically challenged wt and mdx fibers

**Figure 9:**
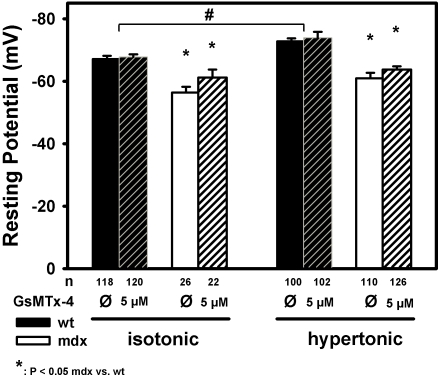
Mechanosensitive channel activity during osmotic challenge does not induce marked membrane depolarisations in mdx fibres. Resting membrane potentials in many intact fibres were recorded by repetitive impalement of whole interossei muscles from wt (black) and mdx (white) mice without enzymatic treatment under isotonic and hypertonic conditions. The contribution of cation influx through mechanosensitive channels to the resting potential when muscles were immersed in either isotonic or hypertonic Ringer solution was assessed by pre-incubation with 5 µM of the spider peptide GsMTx-4, a selective MsC blocker. Although potentials were more depolarised in mdx fibres already under isotonic conditions, this did not increase under hypertonic conditions, nor was there a marked contribution from MsC to depolarisation. n: number of individual potential recording.

In mdx fibers, the increased ECRE frequencies under isotonic and, in particular, after osmotic challenge seem to be a direct consequence of aberrant MsC activity due to the lack of dystrophin. The modulation of the DHPR inhibition on the RyR1 via the α1_S_ II–III loop could be directly or indirectly relieved or alternatively, the RyR1 be activated via depolarisation of the tubular membrane following cation influx through the MsC. In dystrophic muscle, a higher resting [Na^+^] has been described [43]. To test whether mdx muscle were more depolarised than wt following osmotic challenge and whether this was induced by MsC, resting membrane potentials E_m_ were measured in mdx and wt muscle under isotonic and hypertonic conditions, either without or in the presence of the specific MsC blocker GsMTx-4 (5 µM). To obtain recordings that as closely as possible reflect the physiological condition of the muscle fibers, recordings were made in whole muscles exposed from the paw bathed in the appropriate solution. We chose not to record from enzymatically isolated single fibers because the enzymatic treatment may already induce some unspecific damage to the membrane that might readily increase Na^+^ influx unrelated to MsC. Fig. 9 shows the results from such recordings repetitively impaling muscle flaps at random localizations to obtain E_m_ as negative deflections (Methods). In the wt, mean E_m_ was −67 mV under isotonic conditions and significantly more negative under hypertonic conditions (−72 mV), as would be expected for this tonicity increase [56]. Also, the contribution of MsC activity to E_m_ was only marginal. In mdx fibers under isotonic conditions, E_m_ was significantly more depolarised at ∼−57 mV. However, MsC activity only accounted for about 4 mV of this depolarisation (E_m_∼−61 mV in the presence of 5 µM GsMTx-4). Interestingly, under hypertonic conditions, E_m_ was more negative at −61 mV with a similar further repolarisation in the presence of GsMTx-4 as seen under isotonic conditions. This rules out a major contribution from MsC induced depolarisation accounting for a direct activation of the RyR1 by the DHPR as the cause for the markedly increased ECRE frequencies seen during osmotic challenge in mdx fibers.

## Discussion

### ECRE frequency levels and kinetics in healthy, dystrophic and transgenic mini-dystrophin expressing muscle are modulated by external Ca^2+^

We investigated the involvement of mechanosensitive channels (MsC) in uncoupling spontaneous ECRE suppression in osmotically stressed intact single mdx muscle fibers. Our study is the first to confirm some previous results on *‘uncontrolled’* spark activity in mdx fibers in 50 mM Ca^2+^ containing hypertonic solutions [32]. However, under more physiological conditions (2 mM Ca^2+^), there were also some striking differences. First, spontaneous ECRE activity was present in single intact fibers already under isotonic resting conditions, albeit at much lower frequency than following osmotic challenge. This is a very robust finding from several hundred single fibers and contrasts with the finding from Wang et al. [32] of ‘not any spontaneous Ca^2+^ spark activity’ under resting isotonic conditions in an ungiven number of fibers. Present, albeit very low, spark activity was previously documented in native muscle after having been observed in abundance in the embryonic form with a postnatal decline [44]. In fact, our resting ECRE frequencies for wt fibers are very well in the range of the values given by Chun et al. [44] under similar isotonic conditions (their Fig. 2, ∼0.09*10^−4^ µm^2^, as compared to ∼0.15*10^−4^ µm^2^ in the present study, table 1, wt). Resting ECRE activity was lower in wt compared to mdx fibers and low frequency could be restored by transgenic expression of a mini-dystrophin. The increase in ECRE frequency was much larger in mdx fibers than in wt or MinD fibers upon hypertonic challenge in physiological Ca^2+^ containing medium (2 mM Ca^2+^). It was less prominent under hypotonic challenge in wt and MinD fibers, but similar to hypertonic challenge in mdx fibers. The absolute increase showed a clear dependence on external Ca^2+^, e.g. 1.5-fold smaller SSF in mdx fibers in Ca^2+^-free hypertonic solution (table 1). Interestingly, at 50 mM external Ca^2+^, the relative increase in ECRE frequency became smaller in mdx compared to wt fibers suggesting an upper limit for the already markedly increased ECRE activity in mdx fibers. However, ECRE activity, although somewhat modulated by external Ca^2+^, does not completely rely on it. Therefore, the suppression of spontaneous ECRE in resting intact fibers is supposed to be altered by both osmolarity and external Ca^2+^. In contrast to the findings from Wang et al. [32], ECRE frequency changes always showed a distinct time course for activation and inactivation. In particular, the onset for increase in ECRE frequency was consistently shorter in hypertonic medium compared to hypotonic medium for all strains investigated. This is probably due to a larger tubular membrane tension due to large fiber volume changes in hypertonic solutions but minimal associated change in t-system volume, as found from studies using living cells [45], [46]. In addition, it may be partly explained by an increased spontaneous Ca^2+^ release by hypertonicity ‘per se’ that is expected to increase the SR Ca^2+^ concentration, as judged from studies on toad skeletal muscle skinned fibers after addition of 400 mM sucrose or more to the bathing solution as long as intracellular Mg^2+^ concentrations would not rise ([47]; see also argument below). Interestingly, the distance between consecutive rows of transverse tubules remained constant in healthy intact mammalian fibers stretched to sarcomere lengths up to 3.5 µm [42]. This suggests that unsuppressed ECRE seem to rely on increased t-tubular membrane tension induced by mechanical stress rather than structural rearrangement of tubular geometry in intact mammalian muscle, at least in mammals.

Changes in tubular wall tension are likely to activate mechanosensitive channels [20], [48]. These channel activities as well as spontaneous global Ca^2+^ transients were abolished in patch-clamp experiments using MsC blockers [20]. Dystrophic muscle is osmotically more fragile than wt muscle [10] and relative t-tubular tension changes may be larger due to missing tethering of cytoskeleton components during osmotic fiber volume changes. As a result, MsC activity is increased in mdx fibers due to conversion of gating modes [28]. A higher activity of Gd^3+^-sensitive MsC Ca^2+^ channels (MsCa) has been shown to disrupt Ca^2+^ homeostasis in resting mdx EDL fibers [49], a condition that was worsened by chronic exercise of mdx mice due to increased Ca^2+^ influx through leak channels and MsCa [49]. This parallels nicely with our results that increased ECRE activity after osmotic shock in mdx fibers is completely abolished by MsCa blockers. Our study is the first one to link the loss of ECRE suppression in intact osmotically stressed mdx fibers to an increased activity of MsCa, at least to some extent. Besides that, the specific blocker of cation-sensitive stretch-activated channels, GsMTx-4, also potently inhibited osmotic-induced ECRE frequency increases. It has to be pointed out that from our present results it seems that both cation-specific MsC as well as more Ca^2+^ selective MsCa are involved in the control of ECRE inhibition in healthy muscle. So far, there are some speculations about the molecular nature of the channels involved (see below). However, detailed identification and their pharmacological and physiological properties regarding ion sensitivity and drug response in skeletal muscle are still elusive.

Another striking difference to the only previous recording of Ca^2+^ sparks in a very limited set of single mdx fibers (up to 17, [32]) is the inactivation of increased ECRE activity during prolonged osmotic challenge, both hypertonic and hypotonic, in all three genotypes. Most interestingly, we did not see an ‘uncontrolled’ spark activity that would not come down within 15 min of recording time [32], but instead, inactivation occurred. This was consistently faster in mdx fibers compared to wt and MinD fibers under physiological Ca^2+^ conditions (table 2). In order to determine whether the effect of unphysiologically large Ca^2+^ concentrations used in the previous study [32] were responsible for the suggested ‘uncontrolled’ ECRE activity, we repeated such experiments in mdx fibers with 50 mM Ca^2+^ containing hypertonic solution. Very interestingly, the inactivation time constants in these fibers increased three times and approached identical levels to wt and MinD fibres under 2 mM Ca^2+^ containing hypertonic conditions (table 2). At the same time, the peak spark frequency values under our osmotic conditions in mdx fibres (SSF ∼0.1 ECRE per 100 µm^2^) are very similar to the values given by Wang et al. [32] with about 5–7 sparks per frame (their Fig. 2) in both 2 mM and 50 mM Ca^2+^ containing solution (table 2, see also Fig. 2C) in mdx fibres. Therefore, we can convincingly rule out a Ca^2+^ dependent process during maximum ECRE frequency on the inactivation kinetics of ECRE activity. The exact reason why Ca^2+^ spark activity was reported to remain uncontrollably high remains unclear. However, if one looks closer at the ECRE frequency kinetics in the previous study ([32], their Fig. 2C), one can clearly see an inactivation from the initial peak ∼1 min after hypertonic challenge with spontaneous partial inactivation of spark frequency in hypertonic solution to approximately 50% of activity within three minutes. Unfortunately, the authors did not further comment on this [32].

Our study on ECRE activity, containing the most extensive number of intact adult mdx fibers to date, also shows that mini-dystrophin expression successfully restores the suppressed release control under isotonic conditions. This was previously also shown in MinD myotubes [18] and lines up with recent evidence from our lab that mini-dystrophin can functionally replace dystrophin regarding Ca^2+^ homeostasis [15], [16].

In a recent study, the suppression of spontaneous ECRE in intact adult wt fibers was shown to rely on an intact, fully differentiated t-tubular architecture involving the DHPR and RYR1 geometry because spontaneous Ca^2+^ spark frequency increased more than ten-fold in de-differentiating fibers after 5–7 days in culture [50]. Interestingly, the presence of serum added to the culture medium was crucial in relieving ECRE suppression suggesting the involvement of yet unidentified serum factors in ECRE control [50]. From our results, the control over spontaneous ECRE in intact fibers seems to be mediated by MsC and dystrophin modulates its gain upon tubular membrane stretch. The lower MsC activity in wt fibers would explain why ECRE are only rarely found under isotonic conditions in intact fibers [31], [32], [50], [51]. Moreover, this control seems to be largely abolished in mdx muscle. The nature of this ECRE control is still not resolved.

### MsC activity in osmotically challenged mdx fibers does not directly activate ECRE through membrane depolarisation

Wang et al. [32] suggested a Ca^2+^-induced Ca^2+^ release mechanism (CICR) that may be modulated by MsCa. Studies in healthy mammalian muscle largely excluded a significant involvement of CICR during ec-coupling [52], [53]. Although not experimentally verified yet, it has been suggested that suppression of CICR may be relieved in mechanically stressed muscle or in certain myopathies [32], [51]. Alternatively, an increased Na^+^ influx through MsC could also be responsible for a direct voltage-induced activation of the RYR1, as a greater rise in intracellular [Na^+^]_i_ was found in mdx muscle fibers in response to stretch compared with wt fibers [43]. This MsC-induced depolarisation would eventually trigger voltage-mediated ec-coupling apart from an action potential being present. Apart from a putative depolarisation by promiscuous MsC, a local increase in subsarcolemmal Na^+^ concentration was found in young (12–16 week old) mdx *flexor digitorum brevis* muscle fibers, as a result of altered gating properties of Na_v_1.4 channels [54] that could activate the RYR1 and increase ECRE frequency through depolarisation in the resting state ‘per se’. However, the authors did not address the contribution of unspecific cation influx through MsC to the increased subsarcolemmal Na^+^. Also, the resting membrane potentials given by Hirn et al. [54] with similar values around −50 mV in both wt and mdx single fibers are much more depolarised than in our preparation. A possible explanation might be the enzymatic treatment used to isolate single fibers, as resting membrane potentials are known to be more depolarised following collagenase treatment [55]. Also, the fragility of mdx fibres might induce some unspecific leakage with depolarisation during the mechanical isolation procedure, thus possibly contributing to increased cytoplasmic Na^+^ levels secondary to the experimental protocol and not primary to the absence of dystrophin [43]. Our current approach using exposed whole muscle reflects a ‘closest to the physiological state possible’ condition to record E_m_ values [56]. Under these conditions, E_m_ was already about 10 mV more depolarised in isotonic resting mdx fibres. However, even this substantial difference might not fully explain the already twofold increased spark frequencies that we saw in the isotonic mdx fibers. In intact muscle, SR Ca^2+^ release via the ryanodine channel is initiated by the conformational coupling between the DHPR and the RyR1 upon depolarisation that is reflected by DHPR charge movements. However, in a study on intact single fibers, no difference in the sigmoidal voltage distribution of charge movements was observed between wt and mdx muscle [57]. In fact, in the voltage range of E_m_ values observed here, the membrane potential – charge movement curve should still be at the lower knee of the activation curve [57]. Further evidence comes from single toe muscle fibers under voltage clamp conditions that showed almost no SR Ca^2+^ release transients for potentials up to around −55 mV in wt and mdx fibers [58] suggesting that our E_m_ values in mdx fibres under isotonic conditions were still subthreshold for RyR1 opening. Nevertheless, even if there was some activation of RyR1 openings in mdx fibers under isotonic resting conditions, depolarisation clearly cannot account for the marked ECRE frequency increase in mdx fibers subjected to osmotic shock. Similar to a previous study in amphibian muscle, E_m_ values became more negative when increasing the external osmolarity twofold, in accordance with the Nernst-equation [56]. Also, hypersomolar fiber shrinking at ∼650 mosM is expected to increase cytoplasmic ionic strength and Mg^2+^ concentration resulting in hindered SR Ca^2+^ release [47]. These two arguments effectively rule out an explanation for the depolarisation induced increase in ECRE frequencies in mdx fibers. Clearly, MsC were involved because SSF increase was terminated/prevented by Gd^3+^ and streptomycine in osmotically challenged mdx fibers under 2 mM external Ca^2+^ conditions (Fig. 5). However, specifically blocking MsC with the spider toxin GsMTx-4 only resulted in a minor contribution of cationic flux through MsC to the E_m_ values during osmotic challenge reflected by ∼4 mV more repolarised values. A similarly small repolarisation has been previously documented with Gd^3+^ in mdx fibers under isotonic resting conditions [35]. Interestingly, the situation seems to be completely different during eccentric contractions where a large intracellular increase in Na^+^ concentration was found to be mediated by MsC activity in mdx muscle fibers [43]. The tubular forces acting on MsC during lengthening contractions may well exceed those one that appear under osmotic challenge, thus explaining these differences.

### ECRE are modulated by mechanosensitive channels in dystrophic muscle: possible Ca^2+^ dependent and independent pathways

So, if osmotic challenge now resulted in activation of MsC as demonstrated above, and Na^+^ influx under osmotic challenge is probably minor, what else could contribute to increased spontaneous RyR1 openings in mdx fibres? From the considerations in the literature regarding CICR not to be present in mammalian muscle but putatively being unravelled in diseased conditions, we directly ruled out this mechanism, for the first time, in dystrophic muscle by osmotically challenging mdx fibers in Ca^2+^ free external solutions. Under these conditions, ECRE frequencies also substantially increased in osmotically challenged mdx fibers (table 1) although no Ca^2+^ influx through MsC could have occurred ruling out CICR. GsMTx-4 still was able to block this activity also in Ca^2+^ free hypertonic solutions.

Ca^2+^ entering through MsC in mdx fibers bathed in Ca^2+^ containing hypertonic/hypotonic solutions could, however, modulate ECRE activity via second messenger cascades on a slower time scale. Apart from the involved putative mechanisms at the membrane level, it should be noted that a recent study showed a substantial contribution of reactive oxygen species (ROS) to the initiation of spontaneous ECRE following osmotic shock in intact wt muscle fibers that could be traced to the activation of NADPH oxidase (NOX, [51]). These effects may be even downstream to the MsCa induced Ca^2+^ influx suggested in the present study, as the authors showed that Ca^2+^ was needed for substantial NOX activation [51]. This is also suggested by their much larger time interval needed to produce cytosolic Ca^2+^ responses in Ca^2+^ free solutions (∼min), as compared to the faster TTP values in our study (10–30 s, table 2). Thus, such a mechanism would well explain our observation of a positive correlation of peak ECRE frequencies with external Ca^2+^ concentrations, as additional Ca^2+^ influx through MsCa during osmotic challenge in mdx fibers would activate the NOX pathway in addition to the proposed direct MsC-DHPR-RyR1 interaction (see below). Possible candidates for this MsCa channel may be related to the TRP family, as the Gd^3+^ sensitive growth-factor related channel (GRC) is translocated to the sarcolemma by mechanical stress in mdx muscle [59], thus, representing a source for increased Ca^2+^ influx. Likewise, TRPV2 was shown to belong to osmotically sensitive cation channels in murine aortic myocytes [60]. Out of the TRP channel family, five were detected in skeletal muscle from wt and mdx mice (TRPC1, 2, 3 and 6) by RT-PCR and anti-sense repression of TRPC led to a decrease in Ca^2+^ leak channels in dystrophic fibers [61]. However, the drug sensitivity was not assessed and at the current stage, it is not known which TRP isoforms are the targets for the different drugs used [61]. For these reasons, a direct correlation of TRP isoforms to MsC or MsCa is not possible.

Apart from controlling ECRE, we show that the disruption of MsC/MsCa control in mdx muscle also induced global Ca^2+^ wave activity. These Ca^2+^ waves are of interest, as they only appeared in mdx fibres after osmotic challenge at a prevalence of ∼7%. From recordings such as shown in Fig. 8, the wavefront was estimated to propagate at velocities of between 15 µm/s and 35 µm/s which is in the range of propagation velocities for Ca^2+^ waves observed in polarised intact frog muscle fibers following hypertonic challenge [62]. In frog muscle, these waves were consistent with Ca^2+^ induced Ca^2+^ release (CICR). In mammalian muscle, however, CICR does not occur (see above). When analysing our line-scan recordings, we consistently saw a flat front on the Ca^2+^ sparks (not propagating) that is consistent with a concerted opening of channel arrays rather than recruitment by CICR [52] under our hypertonic conditions in mdx fibers. An alternative likely explanation would be represented by an increased SR Ca^2+^ release through RYR1 in dystrophic skeletal muscle where the inhibitory control of spontaneous ECRE by DHPR [63] was somehow relieved by MsC giving rise to store-overload-induced Ca^2+^ release (SOICR), as previously suggested to regularly occur in cardiac muscle RYR2 [64]. So far, not many studies on SR Ca^2+^ content in mdx muscle are available. SR Ca^2+^ reloading and SR Ca^2+^ leak were not different in EDL fibers from wt and mdx mice [65]. However, from rapid cooling contractures, the total SR Ca^2+^ load was estimated to be roughly 40% decreased in mdx diaphragm muscle [66]. More research is needed to address the SR Ca^2+^ load in dystrophic muscle fibers.

### A model for direct MsC-DHPR-RyR1 interaction in muscle

**Figure 10:**
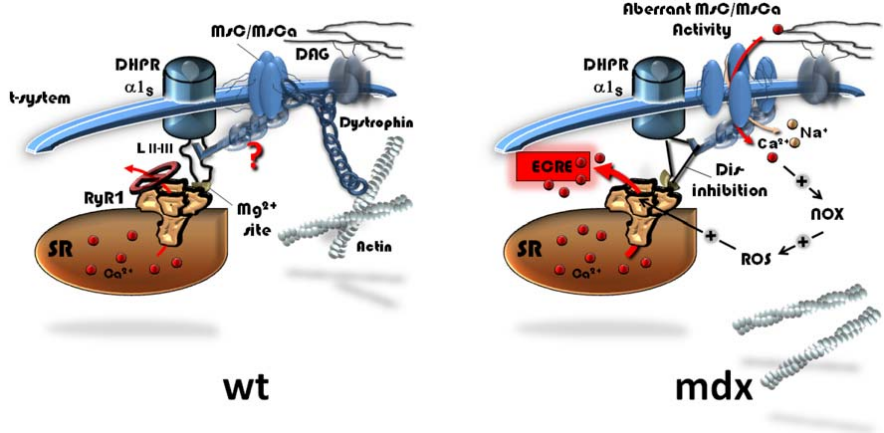
Proposed model of interactions of mechanosensitive channels (MsC/MsCa) with spontaneous ECRE inhibition in wt muscle and relieved inhibition in mdx muscle during osmotic stress. In wt muscle, dystrophin expression is linked to suppressed MsC/MsCa activity probably by directly stabilising them to the membrane scaffold under isotonic and hypertonic conditions. The DHPR α1_S_ subunit exerts an inhibitory effect on the RyR1 Mg^2+^ site via the II–III loop. A direct modulation of this inhibition by mechanosensitive channels is postulated that would not play a major factor in the wt. As a result, under both resting and membrane stress conditions, spontaneous ECRE would be largely suppressed. In the mdx phenotype, stabilisation of MsC/MsCa to the tubule membrane is insufficient in the absence of dystrophin, thus mechanically opening mechanosensitive channels in an aberrantly transducting mode. The putative direct interaction of the latter with the DHPR loop (or other sites) would relief inhibition of the RyR under resting and, more importantly, under membrane stress conditions. ECRE frequency is expected to increase via disinhibited Ca^2+^ release. Depending on additional Ca^2+^ influx through mechanosensitive channels under different external Ca^2+^ containing conditions, peak ECRE frequencies are modulated by Ca^2+^ dependent downstream activation of NOX/ROS pathways that may directly activate ECRE [51]. SR: sarcoplasmic reticulum. DAG: dystrophin-associated glycoprotein complex.

From the discussion above, our results track down the possible mechanisms involved in osmotically induced spontaneous ECRE activity in dystrophin-deficient muscle to a direct interaction of MsC with the inhibitory DHPR-RyR1 loop. We suggest a model (Fig. 10) in which in the MsC is anchored to the inhibitory II–III loop of the DHPR regulating the RYR1 inhibitory Mg^2+^ binding site [67]. Dystrophin would stabilize the membrane scaffold under resting isotonic and osmotic challenge conditions and keep MsC closed as well as minimize the drag on the II–III loop. In dystrophin-deficient muscle, tubular forces are larger due to destabilization of the membrane scaffold that keeps MsC partly open and also increase the drag on the inhibitory II–III loop (see also [20]). This would be even more severe under osmotic challenge, resulting in disinhibition of the RyR1. Depending on the extracellular Ca^2+^ concentration, additional Ca^2+^ influx through mechanosensitive channels would modulate peak ECRE frequencies by the mechanism suggested by Martins et al. [51] through NOX and ROS activation. Regarding the MsC-DHPR-RyR1 interaction, more research is needed to find the exact amino acid site for this molecular interaction for which our study provides the first functional evidence.

### Implications for human DMD muscle and potential drug strategies

How do our results obtained from the mdx animal model translate to the situation in human DMD muscle? The mdx mouse has been confirmed in many studies to be a suitable model to unravel various aspects of the pathophysiological mechanisms in dystrophin-deficient muscle regarding membrane fragility, contractility, inflammatory pathways, Ca^2+^ homeostasis and ec-coupling [12], [16], [17], [19], [20], [68]–[72]. Mdx mice usually show a milder phenotype as compared to DMD patients that is mostly because of efficient regeneration whereas degenerating human muscle is more prominently replaced by fibrotic tissue [73]. Despite their milder phenotype in particular of the limb muscles, specific force remains reduced throughout the life of the animals compared to muscles from wt littermates [69]. Likewise, mdx muscle showed marked uptake of Evans Blue that does not cross the membranes in wt muscle [68]. Interestingly, the degree of sarcolemmal disruption was similar in muscle from mdx mice and DMD patients [68]. Also, by 26 weeks of age, edl muscle from mdx mice showed substantial fibre necrosis, cell infiltration and increased proportion of type I fibres in the mixed soleus muscle [74]. It has been suggested that the mild phenotype in sedentary mdx mice was partly an artefact due to animal keeping conditions that would not encourage active movement, as exercise has been shown to exacerbate the mdx pathology [5], [75]. Studies on human DMD muscle samples certainly would circumvent restrictions imposed by the various animal models. However, the rareness in availability and access to human samples from DMD patients and the difficult handling of such fragile tissue samples explain the rareness of physiological studies on such preparations. Studies on human DMD samples have so far mostly been used for immuno-histochemical and morphological characterisations after fixation and sectioning of tissue (e.g. [76]–[78]). It is also important to understand that physiological studies on human DMD muscle almost exclusively were performed on cultured human myotubes [18], [79], [80] and may not reflect the situation in fully differentiated adult muscle fibers. Human adult muscle has so far, to our knowledge, only been used in one study investigating the contractile properties of vastus lateralis muscle biopsy samples from dystrophic boys after mechanical skinning of single fibers [81]. Studies from intact single fibers of DMD patients are not available at all. Therefore, a direct translation of results from any intact fiber physiology study performed in mdx mice to human muscle is currently only speculative. Despite this constraint, the mdx mouse is still a very useful model in terms of not only studying the disease mechanisms but also to develop gene therapy concepts, like AAV transfer of smaller dystrophin constructs [82] or exon skipping [7], that would otherwise not be available for human trials [83]. Single cell gene therapy in human muscle has so far been only successfully applied ‘ex situ’ in cultured human myotubes [84].

One clinically relevant interpretation of our study would also be that blockers of mechanosensitive channels might prove beneficial on the phenotype in dystrophic muscle if more muscle-selective compounds could be found. The MsC blockers used in our and other studies are mainly used as tools to study cellular reactions on altered channel availability in acute single cell [30], [43] or chronic myotube culture experiments [85]. In such studies, MsC channel blockers were able to correct for stretch-induced short-term damage, like cytoplasmic Na^+^ overload, and partly restored related reductions in force development [43]. It has been suggested that MsC blockers might even protect against muscle damage in the intact mdx mouse [30], although this has never been directly tested. It would, therefore, be most interesting to know whether a chronic application of MsC blockers to mdx animals could improve the apparent phenotype. However, with the blockers currently available, this seems not possible. Gd^3+^ that is also used in some contrast-agents, has the potential to induce nephrogenic systemic fibrosis [86], streptomycine is an aminoglycoside antibiotics with ototoxic potency and GsMTx-4 is a potent spider toxin. Furthermore, aminoglycosides are known to interfere with ribosomes to recognize stop codons and allow a re-translation of a suppressed mutation in a gene [73]. In mdx mice, gentamicin was able to increase dystrophin expression and to improve muscle performance and functionality, however, most probably due to the re-shifts in the dystrophin gene reading frame rather than any blocking effect to MsC (see [73] for review). However, short-term treatment of mdx mice with streptomycine, where side-effects are of less concern, already has proven to protect dystrophic muscle from eccentric contraction induced muscle membrane damage in vivo [87].

### Conclusions

In summary, our study closes the gap from altered MsC activity [20] to impaired Ca^2+^ transients in mdx fibers [19], including now the link to the control of spontaneous ECRE in intact mammalian muscle. Mini-dystrophin expression effectively restores ec-coupling and Ca^2+^ handling and supports the ongoing research for suitable gene transfer strategies in DMD. Another implication of our study is that suitable mechanosensitive channel blockers might be useful in a potential drug therapy setting in DMD.

## Supporting Information

Movie S1 Movie of the Ca^2+^ wave activity in the mdx fibre shown in Fig. 8A. The Ca^2+^ wave is initiated at the one fibre end and spreads with a velocity of approximately 25 µm/s.
(0.54 MB MOV)
